# A model to facilitate self-management of human immunodeficiency virus in students within a university setting and promoting their mental health

**DOI:** 10.4102/hsag.v25i0.1069

**Published:** 2020-09-25

**Authors:** Teolene G. Diedricks, Chris P.H. Myburgh, Marie Poggenpoel

**Affiliations:** 1 Department of Educational Psychology, University of Johannesburg, Johannesburg, South Africa; 2 Department of Nursing Sciences, University of Johannesburg, Johannesburg, South Africa

**Keywords:** HIV, mental health, self-management, university, students

## Abstract

**Background:**

The introduction of antiretroviral treatment (ART) has resulted in people with HIV living longer. Antiretroviral treatment demands a lifelong commitment from patients not only in terms of adherence to the medication but also in relation to lifestyle changes in general. This poses a challenge to a student living with HIV (SLHIV) who only spends a few years at university before entering the workplace and relocating. It also means that the care, support and treatment received at the university will no longer be available to them as these services are only offered to enrolled students. It is imperative for practitioners at universities to help SLHIV effectively manage their illness.

**Aim:**

The aim of the article is to illustrate the process followed to develop a model that could serve as a frame of reference to facilitate the management of HIV as an integral part of the mental health of SLHIV within a university.

**Setting:**

The model is designed for professional practitioners in university settings who support students living with HIV in managing their illness.

**Methods:**

A theory-generative, qualitative, exploratory, descriptive and contextual study design was utilised. The central concept was derived from the experiences of practitioners and SLHIV by conducting individual interviews using appreciative inquiry. The common themes and categories identified in the interviews served as a basis for the identification of the central concept for the study. The process included the identification, definition and classification of the central concept and essential attributes. The conceptual framework was then described. Measures to ensure trustworthiness were also adhered to in the study and approval for the study was granted (Ethical clearance #2014-071).

**Results:**

The central concept was identified as the ‘facilitation of self-management’. It was defined and classified, and these definitions and classifications were used as the basis for the model. Thereafter, the model was described.

**Conclusion:**

The model can be used as a frame of reference to assist SLHIV in effectively managing their illness.

## Introduction

Students living with HIV (SLHIV) are able to live longer because of the introduction of antiretroviral treatment (ART) that has changed HIV into a long-term condition (Cooper et al. 2017). This requires a long-term commitment from the individual diagnosed with HIV to ensure adherence to the medication that helps to suppress the virus, which in turn improves the immune system, making them less vulnerable to other illnesses (Bulsara, Wainberg & Newton-John 2018). However, retaining SLHIV in HIV care remains a challenge (Bulsara et al. 2018; Denison et al. 2015). The loss to follow-up and challenges associated with retention in care is a general problem experienced amongst the youth in colleges as reported in the literature (Minniear et al. 2013; Sutton et al. 2011; Warren-Jeanpiere, Jones & Sutton 2011). Researchers concur that people who were diagnosed whilst they were in college were four times more likely to delay entry into care (Minniear et al. 2013; Sutton et al. 2011; Warren-Jeanpiere et al. 2011).

### Problem statement

The inability to help SLHIV to effectively manage their HIV infection led to practitioners becoming frustrated, which negatively impacted the services delivered to SLHIV. The emotional turmoil, fear of disclosure, stigma, shame and rejection experienced by SLHIV also hampered their ability to seek and continue in care and support services offered by the university.

It was thus necessary to develop a model for practitioners caring for SLHIV in a university to help them to effectively manage their illness and, in doing so, improve the mental health of these students.

### Purpose and objectives

The purpose of the study was to describe and develop a model that could serve as a frame of reference to facilitate the management of HIV as an integral part of the mental health of SLHIV within a university. The steps used to develop the model are described in the following section from the development of the concept to the final model.

## Model development

A model was developed based on theoretical and empirical evidence by applying an inductive, theory-generating research approach. The steps followed in developing the model is described and demonstrated by means of examples from the study.

### Background of the study

A need exists for an HIV practice model to help SLHIV effectively manage their illness, specifically to address their medical, psychological and social needs (Crepaz et al. 2014; Global Network for PLHIV [GNP+] and Joint United Nations Programme on HIV and AIDS [UNAIDS] 2013). This required the study to adopt a student-centred perspective, coupled with an HIV practitioner’s perspective as a point of departure to generate findings to (as far as possible) reflect the experiences of both the SLHIV and HIV practitioner.

### Theoretical approach of the study

The theoretical approach adopted for this study is embedded in the nursing theory, the School of Nursing paradigm (University of Johannesburg 2012), the self-efficacy theory of behaviour change rooted in the social cognitive theory (Bandura 1989b, 1999) and appreciative inquiry (AI), which is embedded in social constructivism where the SLHIV and psycho-educational facilitator construct multiple realities to bring about positive change and create a better future for SLHIV (Kessler 2013). The purpose of the theory for health promotion (University of Johannesburg 2012) is to promote the mental health of SLHIV, and its objective is to mobilise resources for the SLHIV. The SLHIV is viewed as a wholistic being who interacts with the environment in an interactive manner. The HIV practitioner facilitates the mobilisation of resources in the SLHIV’s internal and external environments.

The SLHIV’s internal environment comprises of body, mind and spirit, whereas their external environment refers to the physical, social and spiritual dimensions (University of Johannesburg 2012). The SLHIVs’ interaction with their environment can either enhance or inhibit their mental health. Furthermore, the synergy between the internal and external environments will determine how well the HIV practitioner is able to facilitate the mental health of SLHIV.

The social cognitive theory is a human agency approach that focuses on self-development, self-regulation and self-reflection (Bandura 1989a). Students living with human immunodeficiency virus are viewed as being able to self-reflect, grow, develop and self-regulate their actions and behaviours. The SLHIV can effect change in themselves and their situations through their own efforts. They are not shaped by their environment over which they have no control and have the capability to cope with the challenges related to HIV. The self-efficacy theory further determines how long a person will persist in the face of obstacles and adverse situations (Bandura & Adams 1977). Self-efficacy theory includes determinants of human motivation, affect and action that work together (Bandura 1989a). Students living with human immunodeficiency virus have the capability to persist in the face of setbacks and difficulties related to HIV. This theory was appropriate for the development of a psycho-educational model that promotes mental health as it relates to a psychological phenomenon and has growth and change as core concepts.

### Research design

An inductive, theory-generating approach (Chinn & Kramer 2015), coupled with a qualitative, exploratory, descriptive and contextual research design (Creswell 2014) assisted the researchers in generating theory. The qualitative research design allowed the researchers to interact with both SLHIV and HIV practitioners through open-ended questioning during individual interviews. This enabled the researchers to gain a deeper understanding of experiences of SLHIV and HIV practitioners dealing with SLHIV, which fits well within an exploratory design (Babbie 2017). The descriptive nature of the design assisted the researchers in providing a detailed account of the experiences of SLHIV and HIV practitioners by means of appreciative inquiry, which is well suited with the theory for health promotion (University of Johannesburg 2012). The study is contextual as it refers to the field of HIV practice within a university setting with participants involved in a specific wellness programme.

Finally, the aim of a theory-generating approach assisted the researchers to inductively identify a set of well-defined concepts to help describe the phenomenon (Gray, Grove & Sutherland 2017). Concepts served as the building blocks for theory generation (Walker & Avant 2014) and enabled a broadened application by way of frameworks and models to guide practice (De Vos 2011).

### Ethical consideration

The researchers adhered to the four ethical priciples of autonomy, beneficence, non-malificence and justice throughout the research process of collecting, analysing and reporting of data (Dhai & McQuoid-Mason 2011) were applied. Respect for autonomy was practiced by inviting participants, obtaining written consent for participation in interviews and for interviews to be audiotaped. Participants were free to withdraw participation at any stage; audiotapes were stored by the researchers on a computer, which is password protected; data will be destroyed 2 years after publication of the study; any publications will not contain the names of any of the participants.

Ethical clearance was obtained from a Faculty Research Ethics Committee of an institution of higher education, clearance number 2014–07.

### Trustworthiness

The criteria of credibility, transferability, dependability and confirmability were applied to ensure trustworthiness in this study (Lincoln & Guba 1985). The mentioned criteria were applied during data collection and analysis and findings of this study had been peer-reviewed.

### Method of model development

The four steps of Chinn and Kramer (2015) were applied to develop the model. Figue 1, shows a flow diagram for an overview of the steps.

### Step 1: Concept analysis

Concept analysis was used to identify and classify concepts for model development. It comprised of two phases which are described in the following section.

**FIGURE 1:**
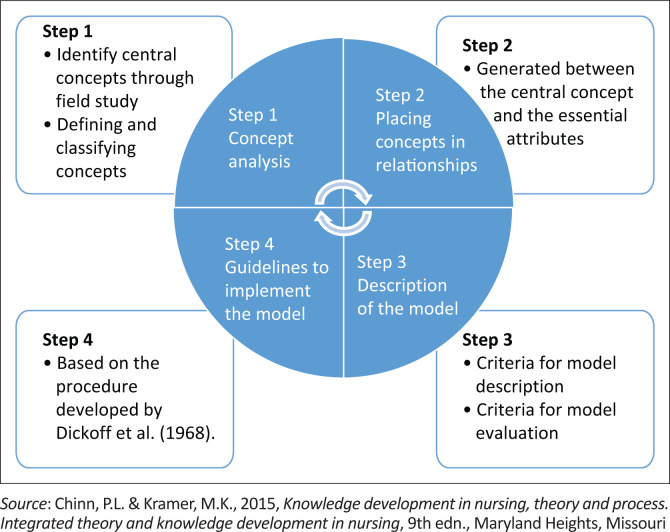
Steps in model development.

#### Phase 1: Identification of the central concept

Appreciative inquiry was used to explore the experiences of HIV practitioners and SLHIV by conducting in-depth individual interviews.

The following components formed part of determining the themes to develop the model:

#### Population and sample

The population included nine HIV practitioners employed in professional services within a clinical, psychological and HIV practice setting, and 10 SLHIVs who were enrolled in the wellness programme at a university in Gauteng, South Africa. Participants were purposefully selected to participate in the study (Babbie 2017). A total of 19 interviews were held. The student participants’ ages ranged from 19 to 24 years and included four males and six females who were enrolled in the wellness programme for at least 6 months. Practitioners had vast experience dealing with SLHIV, which varied between a maximum of 15 years to a minimum of 3 years. Data saturation was achieved after eight interviews were conducted with practitioners and nine interviews were conducted with SLHIV.

#### Data collection

In-depth individual interviews were conducted because of the sensitive and confidential nature of this study. The researchers developed the interview schedule and piloted it to identify potential problems related to the research design and formulation of questions (Gray et al. 2017). Problems with some of the questions were identified and the researchers adapted it. Once ethical clearance was received, the researcher contacted the HIV practitioners in professional services, who were involved in the wellness programme at the university under study. Appointments were scheduled during standard staff meetings to limit disruptions to services. The nature of the study was explained to HIV practitioners and consent forms and participant information sheets were distributed. Interested HIV practitioners were requested to complete the consent forms and to email it to the researcher’s personal email that is password protected.

The researcher scheduled interviews with HIV practitioners who consented, at a time and venue that was suitable to individual practitioners. Human immunodeficiency virus practitioners assisted in the enrolment of prospective student participants for the study. The sampling criteria with the accompanied consent form and participant information sheet were explained to HIV practitioners. Students who were willing to participate in the interviews were required to leave a signed consent form with the HIV practitioner or email the signed consent form to the researcher’s personal email that is password protected. Completed consent forms were collected 1 week later. Participants were contacted and the nature of the study was explained to them. Individual interviews were scheduled at a time and venue suitable for students to ensure confidentiality and minimal disruptions to their studies. A quiet office on campus was used to facilitate audiotaping of interviews. Individual interviews lasted from 45 min to an hour.

The following questions were posed to research participants during individual interviews. See Table 1.

**TABLE 1:** Questions posed to research participants.

4-D Model elements of AI	HIV practitioner	SLHIV
Discovery	Tell me about dealing with SLHIV?	Tell me about dealing with living with HIV?
Dream	How can you deal differently with SLHIV?	How can you deal differently with living with HIV?
Design	What should be included in dealing with SLHIV?	What should be included for a person dealing with HIV?
Destiny	How could it be achieved?	How could it be achieved?

*Source*: Diedricks, T.G., Myburgh, C.P.H. & Poggenpoel, M., 2018, *A model to facilitate the mental health of students living with HIV and practitioners caring for them*, University of Johannesburg

AI, appreciative inquiry; HIV, human immunodeficiency virus; SLHIV, student living with human immunodeficiency virus.

The researcher wrote down feelings and experiences that occurred during the interview process. These personal field notes were taken at the end of each individual interview. This process ensured that the researcher’s personal HIV stories were bracketed before the data analysis happened. Observational notes were based on non-verbal cues detected during the interview process. Descriptive and reflective field notes were compiled during and after each individual interview (Creswell 2013). Methodological notes were written in an attempt to remind the researcher about slight changes for inclusion in the next interview session. Theoretical notes were the researcher’s initial thoughts on the possible themes that could emerge and topics that were later brought into the literature review (Babbie 2017). Appreciative inquiry was employed during interviews by applying the 4-D model (Bushe 2011; Cooperrider 2017; Kessler 2013).

#### Data analysis

Open coding was used to conduct the data analysis (Creswell 2013). Interviews for HIV practitioners and students living with HIV were transcribed separately and field notes for each were typed. The data were coded by categorising the data and labelling the themes and categories for HIV practitioners and students, respectively, with descriptive terms generated. An independent coder was consulted to verify the themes that were generated (Creswell 2013). A consensus meeting was held between the researchers and the independent coder to reach consensus on the identified themes. Once final agreement on the themes was reached, the researchers arranged a meeting with HIV practitioners who participated in the individual interviews to discuss the themes. A separate meeting was also held with individual student participants who were available. Participants had an opportunity to discuss their impressions of the themes and verify that the themes were a true reflection of the experiences discussed in the individual interviews. A literature control was performed to relate the findings to the current HIV dialogue in the literature (Havenga, Poggenpoel & Myburgh 2014).

The themes and categories identified for students and HIV practitioners, respectively, were finally compared to determine common themes and categories shared between them. The researchers applied inductive reasoning for this process. An independent coder was consulted and consensus was reached. Common themes and categories identified served as the basis for describing and classifying the central concept for the model. Common themes are summarised in Table 2.

**TABLE 2:** Common themes identified during individual interviews with student living with human immunodeficiency virus and practitioners dealing with SLHIV.

Themes	Central concept
**Theme 1:** It is tough being diagnosed with HIV.	Facilitation of self-management
**Theme 2:** Experience in disclosure-guidance leads to a positive mind set.	
**Theme 3:** Experience in holistic treatment at accessible clinics with well-qualified staff is essential.	
**Theme 4:** Experience in education and policy involvement benefit SLHIV.	

*Source*: Diedricks, T.G., Myburgh, C.P.H. & Poggenpoel, M., 2018, *A model to facilitate the mental health of students living with HIV and practitioners caring for them*, Auckland Park, Johannesburg

HIV, human immunodeficiency virus; SLHIV, student living with human immunodeficiency virus.

The researchers applied inductive reasoning to combine the themes from interviews into a general concept that could be transferable to a broader HIV population. They reflected on the central story and common themes identified during interviews with participants. Through this process, the central statement identified from the findings was *facilitation of self-management*, and the central concepts were *facilitation* and *self-management.* Online dictionaries and subject definitions in the fields of education, nursing and psychology were used to define *facilitation* and *self-management.*

#### Phase 2: Defining and classifying concepts for the model

By combining the essential and related attributes of the dictionary and subject literature definitions, a final definition was formulated by the researchers. Essential attributes created conceptual meaning for the concepts *facilitation* and *self-management.* Table 3 provides a summary of the attributes for the concept *self-management* as derived from the dictionary and subject literature definitions.

**TABLE 3:** Essential attributes of self-management.

Concept	Essential attributes	Related attributes
**Self-management**	**Control**	Taking responsibility for own health, behaviour and well-being
	**Ability**	Developing and using cognitive, behavioural and emotional strategies
	**Active participation**	Management of infection and related processes Encompasses physical, psychological, social, spiritual, existential and system-related processes Partnering with a psycho-educational facilitator

*Source*: Diedricks, T.G., Myburgh, C.P.H. & Poggenpoel, M., 2018, *A model to facilitate the mental health of students living with HIV and practitioners caring for them*, Auckland Park, Johannesburg

Table 4 provides a summary of the attributes for the concept *facilitation* as derived from the dictionary and subject literature definitions.

**TABLE 4:** Essential attributes of facilitation.

Concept	Essential attributes	Related attributes
**Facilitation**	**Process**	Guided and built on a trusting relationship between the facilitator and those engaged.
	**Enable actions**	To make it easier for individuals to feel in control and empowered Take ownership
	**Change**	In the way individuals think and act In their ability to reflect on everyday practices
	**Dynamic and interactive**	Creation of a positive environment Mobilisation of resources Bridging of obstacles

*Source*: Diedricks, T.G., Myburgh, C.P.H. & Poggenpoel, M., 2018, *A model to facilitate the mental health of students living with HIV and practitioners caring for them*, Auckland Park, Johannesburg

The identified and defined essential attributes of the concepts related to the central concept were used to define *facilitation of self-management.*

Table 5 summarises the essential attributes for *facilitation of self-management.*

**TABLE 5:** Essential attributes-facilitation of self-management.

Concepts	Essential attributes
Self-management	Control Ability Active participation
Facilitation	Process Enable action Change Dynamic and interactive

*Source:* Diedricks, T.G., Myburgh, C.P.H. & Poggenpoel, M., 2018, *A model to facilitate the mental health of students living with HIV and practitioners caring for them*, Auckland Park, Johannesburg

The final step was to develop a model case to identify what was experienced in the central concept and what the central concept did not reflect. The essential attributes, which form part of the definition of the central concept, were reflected in the model case as described next.

The *facilitation of self-management* by the HIV practitioner refers to a dynamic interactive process between the psycho-educational facilitator and the SLHIV. This process involves enabling actions by the psycho-educational facilitator that lead to change in the SLHIV. The SLHIV takes control through active participation in partnership with the psycho-educational facilitator to unlock the SLHIVs ability to develop and use cognitive, behavioural and emotional strategies to deal with their infection (Diedricks et al. 2018).

**FIGURE 2:**
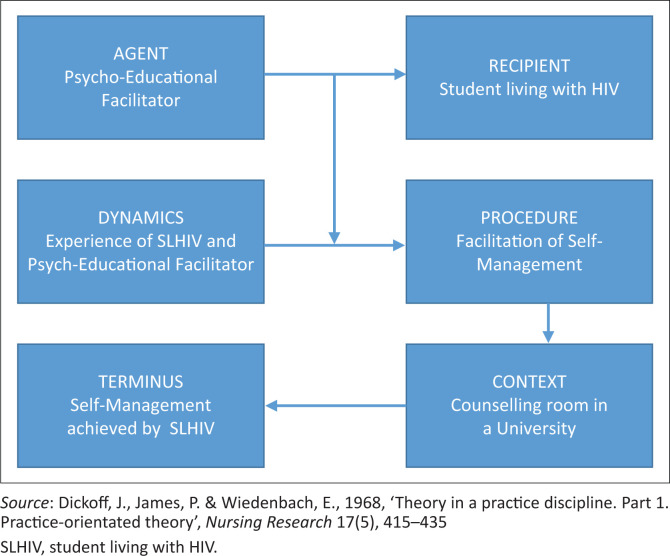
Classification of concepts.

Concepts were classified by means of Dickoff, James and Wiedenbach’s survey list (1968) as illustrated in Figure 2. This survey list assisted in identifying important elements as basis for model development such as agent, recipient, dynamic, procedure, context and terminus:

1. *The agent* is the psycho-educational facilitator who can facilitate the process towards active participation and management of their HIV infection for SLHIV.
2. *The recipient* is the SLHIV who is unable to achieve active participation in the management of their HIV infection.
3. *The context* represents a counselling room that is situated in a university within a wellness programme that is based in a health clinic, counselling centre and HIV Centre.
4. *The dynamics* refer to the experiences of the psycho-educational facilitators and SLHIV who are responsible for the initiation of the process of change. Students living with human immunodeficiency virus portray initial experiences of turmoil and emotional distress when diagnosed with HIV, which disrupts their lives and negatively affects their mental health. Living with HIV is a life-changing event that requires major physical, psychological and social adjustments in order to deal effectively with the HIV diagnosis. During the process, the ability of the SLHIVs to self-manage their HIV infection emerged, paving the way to deal with living with HIV. The initial experience of psycho-educational facilitators is that it is challenging to deal with SLHIV as HIV is complex in nature. However, the passion and commitment portrayed by psycho-educational facilitators, in spite of the challenges, paves the way for the psycho-educational facilitator to help SLHIV make adjustments and adapt to living with HIV. Both SLHIV and psycho-educational facilitators are willing to participate on a journey of growth and change, which is the force behind this process (Diedricks et al. 2018).
5. *The terminus* is students’ self-management of HIV (Diedricks et al. 2018).
6. *The procedure* is the facilitation of self-management (Diedricks et al. 2018). Facilitation of self-management takes place through a dynamic interactive process between the psycho-educational facilitator and the SLHIV. It involves enabling actions by the psycho-educational facilitator that lead to change by the SLHIV. The SLHIV takes control through active participation in partnership with the psycho-educational facilitator to unlock the SLHIVs ability to develop and use cognitive, behavioural and emotional strategies to deal with their infection (Diedricks et al. 2018).

#### Step 2: Placing concepts into relationships

Relationship statements show the connection between the concepts of the model. It served as the basis for the structure of the model as a frame of reference to facilitate the mental health for SLHIV in a university. Relationship statements were generated between the central concept and the essential attributes (Chinn & Kramer 2015). The following relationship statements illustrate this process (Diedricks et al. 2018):

1. The HIV practitioner interacts in a dynamic and interactive process with the student to enable actions that lead to change in the SLHIV.
2. The SLHIV, through *active participation* and in partnership with the psycho-educational facilitator, takes *control* of living with HIV.
3. Through a partnership with the HIV practitioner, the SLHIV unlocks his or her ability to develop and use cognitive, behavioural and emotional strategies to deal with the HIV infection.

#### Step 3: Description of the model

**FIGURE 3:**
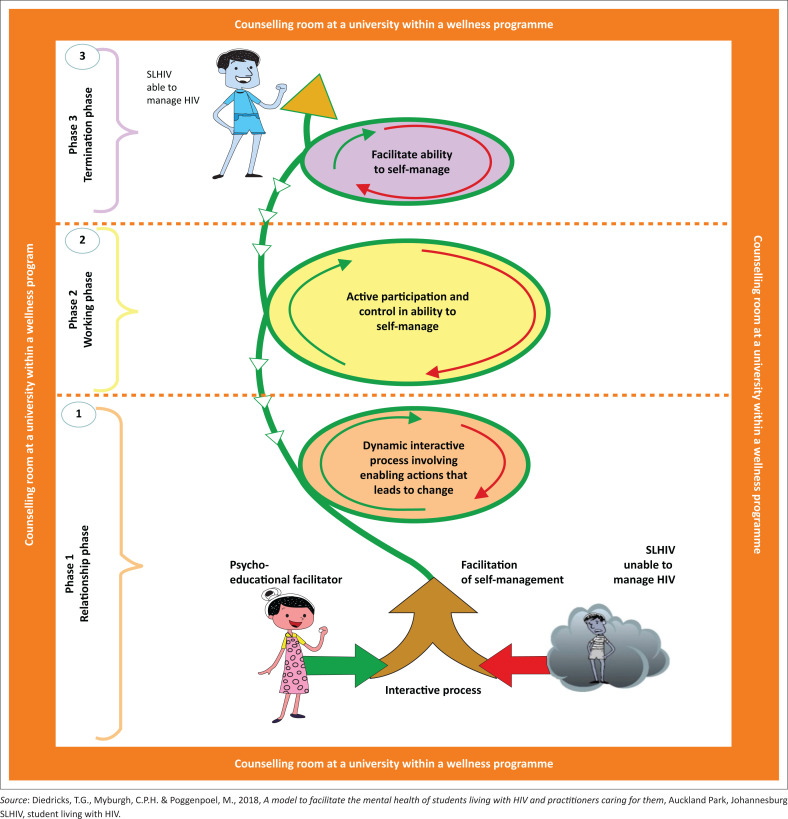
A model to facilitate self-management of student living with human immunodeficiency virus.

An overview of the structure and process of the model is described. See Figure 3.

**Structure and process of the model:** The structure of the model is based on Chinn and Kramer’s concept analysis (2015), specifically referring to the relationship between concepts as illustrated in Step 2: Placing concepts into relationships of this article. The process of the model illustrates the phases that need to be followed in order to attain self-management by the SLHIV. Both the structure and process of the model function interdependently and will be discussed jointly. Figure 3 illustrates the structure and process of the model.

The green upward spiral illustrates the change of the SLHIV from being unable to manage HIV to being able to manage HIV. This green colour depicts the continuity of growth, renewal and restoration (colorpsychology.org 2014). The change is driven by dynamic interactions between the SLHIV and the psycho-educational facilitator, which is illustrated in three distinct phases each relating to actions. The three phases are discussed in the following section.

**Context of the model:** The orange border represents the counselling room in a university within a wellness programme. The counselling room is a safe space where the facilitation of self-management between the psycho-educational facilitator and the SLHIV occur. The orange colour symbolises warmth and offers emotional strength in difficult times (Diedricks et al. 2018). The orange colour assists in recovery from grief that helps the SLHIV to bounce back from disappointment and despair (colorpsychology.org 2014). The interaction between the psycho-educational facilitator and SLHIV is characterised by the establishment of trust, mutual respect, unconditional acceptance, openness and honesty (Diedricks et al. 2018).

**Relationship phase:** Phase 1 comprises two arrows moving from the psycho-educational facilitator towards the SLHIV. These arrows illustrate the initial interactive process between the psycho-educational facilitator and the SLHIV. During the initial interactive process the SLHIV is unable to manage his or her HIV and presents with emotional distress, which severely affects the physical, psychological and social aspects of their lives. The dark grey cloud and small grey figure of the SLHIV inside the cloud illustrate the lack of energy displayed by the SLHIV because of the turmoil and emotional distress that the SLHIV is initially experiencing. The psycho-educational facilitator provides a safe and trusting environment for the SLHIVs who take a conscious decision to enter into a partnership with the psycho-educational facilitator towards the facilitation of self-management. The psycho-educational facilitator is dressed in pink, which shows the kind, empathetic and nurturing nature of the facilitator. The facilitator creates a positive environment in order for the dynamic and interactive process to take place between themselves and the SLHIV. The two arrows merge and the dynamic interactive process that entails enabling actions that will lead to change begins. The psycho-educational facilitator’s participation continues as depicted by the green arrow inside the first spiral and the SLHIV’s participation continues as illustrated by the red arrow inside the first spiral. The participation from the psycho-educational facilitator is more compared with the SLHIV as seen by the length of the arrows inside the first spiral.

**Working phase:** The role of the psycho-educational facilitator is to facilitate active participation and control to unlock the SLHIVs’ ability to self-manage their HIV infection. The psycho-educational facilitator uses psycho-educational techniques to facilitate cognitive, emotional and behavioural strategies to enable change towards an increased ability to self-manage and enhancement of mental health. The SLHIVs actively start to become aware of self-management and start to use it in their daily lives. This increases their ability to control their HIV infection. As the SLHIVs start to use self-management on their own, the psycho-educational facilitator starts to fade out as depicted by the short green arrow on the left hand side inside the second spiral. The working phase represents the largest spiral as most time is spent in this phase. The inside of the spiral is coloured yellow that indicates the clarity of thought, newly acquired knowledge and finding new ways of doing things by the SLHIV, which leads to a sense of self-worth and promote mental health (colorpsychology.org 2014). The facilitation process is flexible and allows for the SLHIV to sometimes move back into a previous phase and then proceed in a forward movement towards self-management again. This relapse process is indicated as small white arrows in the model; it starts outside the termination phase on top and moves downwards towards the working phase. The relapse is a temporary process and is deemed normal considering the complex nature of HIV.

**Termination phase:** The SLHIV’s ability to self-manage is illustrated by the long red arrow inside the spiral, which occupies most of the space inside the spiral. The termination phase is the smallest spiral in the facilitation process, which illustrates that the SLHIV is able to self-manage their HIV without the guidance of the psycho-educational facilitator. The SLHIVs’ ability to self-manage is further illustrated by the long red arrow inside the spiral, which occupies most of the space inside the spiral. The SLHIV is able to overcome obstacles by using self-management. The skills and knowledge of self-management have been successfully transferred to the SLHIV and they are able to use self-management in the future when they are faced with challenges. The inside of the spiral is shaded lilac, which portrays mental balance and stability (colorpsychology.org 2014) that has been achieved by the SLHIV through the process of self-management. The role of the psycho-educational facilitator minimises as guidance is no longer needed, which is illustrated by the short dotted green arrow. Students living with human immunodeficiency virus have now integrated HIV in all dimensions of their daily lives.

The green colour continues after the termination phase. This depicts the continuity of growth, renewal and restoration (colorpsychology.org 2014) for the SLHIV who is able to self-manage. The colour of the SLHIV changed from dark grey in the initial interactive process of the relationship phase to light blue in the termination phase which illustrates the SLHIV becoming more responsible, confident and able to take control in managing his or her HIV infection (colorpsychology.org 2014). The facilitation process ends in the termination phase. However, the SLHIV continues on the self-management path as indicated by the big golden arrow, which symbolises a higher level of understanding and wisdom (colorpsychology.org 2014) that the SLHIV has acquired in the self-management process. The SLHIV continues with the process of self-management beyond the termination phase, and self-management becomes a way of life for the SLHIV.

**Evaluation of the model:** The model was evaluated by means of Chin and Kramer’s critical reflection (2015): how simple is this model? how clear is this model for you? how accessible is this model for you? how important is this model? how general is this model? Seven academic panel experts with vast experience in model development participated in the evaluation process. The following feedback was received from panellists (Diedricks et al. 2018):

##### 1. How simple is this model?

The feedback received from the panel of assessors was that the model could be improved to be simpler. The following comments were received:

‘The essential criteria is not reflected in the working phase.’

‘The essential criteria of self-management are not clear in the model.’

‘Like the use of the colour green for the psycho-education facilitation.’

‘Like the red colour portrayed as will to survive. It’s of great significance.’

‘Could be simpler.’

‘Include initiation as part of the relationship phase.’

The simplicity of the model was improved according to the feedback received.

##### 2. How clear is this model for you?

The clarity of the model in terms of its structure was unclear. The following comments were received from the assessors:

‘Greater clarity needed between the use of the cone, arrows and triangle.’

‘Adjust the spiral and make it broader.’

‘Unclear where the process for the facilitator and the process for the student ends.’

‘Content of phases not clear.’

‘Roles of student and facilitator not clear.’

The recommendations received from the assessors concerning the structure and concept alignment was implemented and the model was improved accordingly.

##### 3. How accessible is this model for you?

The feedback revealed that the model is very useful and critical for the mental health of SLHIV. Comments received from the panel of assessors reflected the following:

‘When model is adjusted to be simpler and clearer, it will be accessible.’

‘Self-empowerment is critical in mental health for SLHIV.’

##### 4. How important is this model?

The model was perceived as important and relevant in the sector it was applied, namely higher education. The following comments were received:

‘Really important.’

‘Most suitable in the learning environment.’

‘Important because of the high rates of HIV at universities.’

##### 5. How general is this model?

It was evident from the feedback received that the model could be widely applied within the health and education contexts. The following comments were received:

‘Can be transferred to all situations where a person has been diagnosed with a chronic disease.’

‘Can be applied in similar set-ups or other set-ups where growth and support would be needed.’

#### Step 4: Guidelines to implement the model

Guidelines were developed to help the psycho-educational facilitator in a university setting to facilitate self-management of HIV in SLHIV and enhancing their mental health. It is based on the procedure described by Dickoff et al. (1968), which is aligned with the structure and process of the model. Guidelines were designed for the three phases of the model with detailed objectives and related actions to guide the psycho-educational facilitator. The researchers were guided by the six broad stages of self-management as outlined in Rotheram-Borus et al. (2012). These stages are aligned with the journey of change towards self-management of the SLHIV during the working phase. Motivational interviewing (MI) formed the basis for the relationship phase guidelines. Motivational interviewing is a technique that can help the psycho-educational facilitator to enable actions that will lead to change (Elwyn et al. 2014).

## Conclusion

This article illustrated the development of a model in relation to a specific HIV practice-related phenomenon. The purpose of this model is the promotion of SLHIV’s mental health by the HIV practitioner through the facilitation of self-management. The model applies to SLHIV enrolled in a wellness programme in a specific university and practitioners employed at a university who work in professional services in a clinical, psychological and HIV practice setting. The implementation of the model to facilitate self-management for SLHIV could further strengthen and promote psycho-educational support in HIV practice.
